# Fish TOLLIP manipulates ATG5 for autophagic degradation of STING to attenuate antiviral interferon responses

**DOI:** 10.1371/journal.ppat.1013512

**Published:** 2025-09-12

**Authors:** Meng-Ze Tian, Yang-Yang Wang, Bao-Jie Cui, Xiao Xu, Chu-Jing Zhou, Can Zhang, Zhuo-Cong Li, Meng-Qian Hong, Na Xu, Dan-Dan Chen, Long-Feng Lu, Shun Li

**Affiliations:** 1 Key Laboratory of Breeding Biotechnology and Sustainable Aquaculture, Institute of Hydrobiology, Chinese Academy of Sciences, Wuhan, China; 2 University of Chinese Academy of Sciences, Beijing, China; 3 College of Fisheries and Life Science, Dalian Ocean University, Dalian, China; 4 Laboratory for Marine Biology and Biotechnology, Qingdao Marine Science and Technology Center, Qingdao, China; 5 Key Laboratory of Aquaculture Disease Control, Ministry of Agriculture, Wuhan, China; Colgate University, UNITED STATES OF AMERICA

## Abstract

While robust interferon (IFN) responses in fish are critical for viral clearance, dysregulated signalling can trigger detrimental hyperinflammation, necessitating precise immunoregulatory mechanisms. This study identified Toll-interacting protein (TOLLIP) as a pivotal negative regulator of IFN production in grass carp *(Ctenopharyngodon idella)*. Upon grass carp reovirus (GCRV) infection, TOLLIP expression increases significantly in tissues and cells. Furthermore, TOLLIP overexpression reduced GCRV- and polyinosinic-polycytidylic acid (poly I:C)-induced IFN expression, whereas *tollip* knockdown increased the cellular IFN production capacity. TOLLIP subsequently binds and degrades STING. Further mechanistic studies revealed that TOLLIP degrades STING in a dose-dependent manner via an autophagy-lysosome-dependent pathway. Interestingly, autophagy-related protein 5 (ATG5) was found to interact with TOLLIP and reduce TOLLIP-mediated STING degradation after *atg5* knockdown. In addition, TOLLIP attenuated STING-driven IFN activation and compromised antiviral efficacy. These findings demonstrate that fish TOLLIP plays a specialized regulatory role in antiviral innate immunity, balancing immune defence with homeostasis maintenance.

## Author summary

To prevent inflammatory consequences from dysregulated signalling, IFN responses require tightly regulated immunomodulatory mechanisms. Our study revealed that teleost TOLLIP acts as a critical negative regulator of IFN production by directly interacting with STING to promote its degradation through the autophagy-lysosome pathway, with ATG5 participating synergistically in this process. These findings not only elucidate the novel regulatory function of TOLLIP in cellular immune responses in fish but also provide new insights into the evolutionary complexity of IFN regulatory mechanisms in vertebrates.

## Introduction

The innate immune system serves as the host’s primary defence mechanism against viral invasion through the detection of pathogen-associated molecular patterns (PAMPs) by pattern recognition receptors (PRRs) [1,2]. Among these receptors, retinoic acid-inducible gene 1 (RIG-I)-like receptors (RLRs), comprising three members—RIG-I, melanoma differentiation-associated gene 5 (MDA5), and laboratory of genetics and physiology 2 (LGP2)—play pivotal roles in initiating antiviral immune responses [3]. Upon recognition of viral RNAs, RIG-I or MDA5 recruits mitochondrial antiviral signalling protein (MAVS, also known as VISA, IPS-1, or Cardif) to form signalling platforms, which subsequently activate downstream effectors, including stimulator of interferon (IFN) genes (STING/MITA/ERIS/MPYS) and TANK-binding kinase 1 (TBK1) [4–7]. This cascade culminates in the phosphorylation and nuclear translocation of IFN regulatory factors 3/7 (IRF3/7), driving IFN production and the expression of IFN-stimulated genes (ISGs) [8,9]. In fish, evolutionary conservation of the RLR pathway has been demonstrated through functional studies [10]. For example, zebrafish (*Danio rerio*) MAVS plays a central role in the induction of IFN responses against RNA and DNA viruses [11]. TBK1 in grass carp (*Ctenopharyngodon idella*) is regulated by multiple functions and maintains immune homeostasis through dynamic modulation of signalling intensity [12]. The conserved role of STING in fish antiviral immunity is exemplified by its critical function in crucian carp (*Carassius auratus L.*) [13]. These findings indicate that RLR signalling pathways are likely to be conserved in fish [14].

Although IFN production constitutes a cornerstone of antiviral immunity, dysregulated IFN signalling is associated with immune hyperactivation and autoimmune pathogenesis [15]. As a central hub of innate immunity, STING requires precise regulation to balance antiviral efficacy with homeostatic control. Emerging evidence reveals a multilayered regulatory network involving ubiquitination and protein interactions that restrain STING activity [16]. For example, ring finger protein 5 (RNF5) terminates STING signalling by mediating K48-linked polyubiquitination and subsequent proteasomal degradation [17]. Pellino E3 ubiquitin protein ligase (PELI2) binds phosphorylated STING to attenuate IFN-β production [18]. In fish, STING has a conserved structural architecture analogous to that of mammalian homologs, comprising three functional domains: an N-terminal transmembrane (TM) domain anchoring it to the endoplasmic reticulum (ER) membrane, a central cyclic dinucleotide (CDN)-binding domain responsible for cytosolic pathogen sensing, and a C-terminus (CTT) as a linear signalling hub [18–21]. Emerging evidence highlights the pivotal role of STING in antiviral immunity across diverse fish species, underscoring the need for stringent regulatory networks to balance effective viral clearance with the prevention of immunopathology. For example, the zebrafish transcript variant of NOD-like receptor X1 (NLRX1-tv4) catalyzes the K48-linked polyubiquitination of STING by recruiting the E3 ubiquitin ligase RNF5, eventually dampening IFN responses [22]. Zebrafish cytochrome P450, family 19, subfamily A, polypeptide 1a (Cyp19a1a) targets STING for autophagic degradation by recruiting ATG14, eventually blocking IFN production [23]. Grass carp cGAS-like gene (cGASL) reduces the TBK1-induced phosphorylation of STING to regulate IFN production [24]. Despite these advances, the regulatory mechanisms governing STING activity in grass carp remain poorly characterized.

Autophagy, an evolutionarily conserved homeostatic mechanism in eukaryotes, serves as a critical catabolic pathway responsible for clearing cellular constituents, including protein aggregates, damaged organelles, and intracellular pathogens. This process involves the formation of double-membrane autophagosomes that subsequently fuse with lysosomes to facilitate cargo degradation [25]. Multiple autophagy proteins are involved in the autophagy process, among which the Beclin1 and autophagy-related protein 14 (ATG14) participates in the initiation stage, ATG5 functions during the extension period, SQSTM1 (P62) can recognize substrates, and RAS-associated protein (RAB5A/7b) participates in different stages of vesicle transport to lysosomes [25–28]. Emerging evidence indicates that autophagy exhibits remarkable substrate specificity, selectively targeting distinct cellular components through cargo receptors. Mechanistically, key cargo receptors, including optineurin (OPTN), p62, NBR1 autophagy cargo receptor (NBR1), and toll-interacting protein (TOLLIP), selectively recognize their targets and bind to ATG8 family proteins on phagosomes via their LC3-interacting region (LIR) motif, thereby supporting cargo transport to the phagophore for degradation [29,30]. Accumulating evidence from cross-species studies reveals that selective autophagy serves as an evolutionarily conserved regulatory mechanism for attenuating antiviral signalling pathways [31]. For example, human Tetherin suppresses IFN responses by orchestrating NDP52-mediated selective autophagic degradation of MAVS [32]. Similarly, UNC93B1 attenuates the cGAS-STING signaling pathway by autophagy-lysosome degradation of STING [33]. Evolutionary conservation of this regulatory paradigm was further demonstrated in fish; zebrafish transmembrane protein 47 (TMEM47) facilitates simultaneous autophagic degradation of both MAVS and STING, thereby impairing antiviral defences [34]. Importantly, this recurring mechanism across vertebrates highlights the critical need to investigate whether analogous regulatory networks exist in economically significant aquaculture species such as grass carp, where autophagy-mediated immune modulation remains underexplored [35].

TOLLIP functions as a multifunctional adaptor protein that modulates diverse cellular signalling pathways. It is composed of a target of the Myb1 (Tom1)-binding domain (TBD) at the N-terminus, a conserved core domain 2 (C2) domain containing two LIRs at the center, and a coupling of ubiquitin to the ER degradation (CUE) domain at the C-terminus [36]. TOLLIP critically regulates inflammatory signalling cascades, orchestrates autophagic processes, and facilitates vesicular trafficking mechanisms [36–38]. For example, TOLLIP inhibits toll-like receptor 2/4 (TLR2/4)-triggered NF-κb signalling by suppressing the phosphorylation and kinase activity of IL-1 receptor-associated kinase (IRAK) [39]. TOLLIP specifically stabilizes STING, which is important for maintaining immune homeostasis in tissues [40]. Compared with those in higher vertebrates, *tollip* genes remain characterized in relatively few fish species [41]. Phylogenetic conservation is demonstrated by teleost TOLLIP orthologs (e.g., grass carp) retaining 76% amino acid identity with human counterparts and preserving all canonical functional domains (TBD: aa 1–53; C2: aa 54–172; CUE: aa 231–273). Mechanistically, the large yellow croaker (*Larimichthys crocea*) TOLLIP functionally mediates the suppression of myeloid differentiation factor 88 (MyD88)-mediated NF-κB activation [42]. In Japanese eel (*Anguilla japonica*), TOLLIP functions as a potent negative modulator of the MyD88-dependent TLR signalling pathway during viral and bacterial infections [43]. Furthermore, viral infection induces cytoplasmic colocalization and functional interactions between TOLLIP and IRAK1 in grass carp [44]. There are critical knowledge gaps regarding the immunoregulatory capacity of teleost TOLLIP, especially its potential crosstalk with the RLR-mediated antiviral signalling axis.

In this study, grass carp reovirus (GCRV) infection potently induced both autophagic flux and IFN responses, accompanied by the concomitant upregulation of the selective autophagy receptor TOLLIP. Mechanistic investigations demonstrated that TOLLIP binds STING and recruits ATG5 to facilitate its selective autophagic degradation, thereby suppressing IFN expression. Collectively, these data demonstrate that TOLLIP serves as an IFN-negative regulator to maintain the homeostasis of cellular IFN responses by targeting STING during GCRV infection.

## Materials and methods

### Ethics statement

The fish experiments in this study were conducted under the guidance of the European Union Guidelines for the Handling of Laboratory Animals (2010/63/EU) and approved by the Ethics Committee for Animal Experiments of the Institute of Hydrobiology, Chinese Academy of Sciences (No. 2025–068). The entire study adhered to all relevant ethical regulations.

### Fish, cells, and viruses

Mature grass carp individuals 4 months after hatching (10 ± 2 cm, 8 ± 2 g) were selected for this study and were provided by the Liang Zi Hu Experimental Base of the Institute of Hydrobiology. In accordance with ethical requirements and national animal welfare guidelines, all experimental fish were required to undergo a two-week acclimatization period in the laboratory, and their health were assessed prior to the commencement of the study. Only healthy fish were used for scientific research, and all infection experiments were completed within 30 min.

*Epithelioma papulosum cyprini* (EPC) cells and *Ctenopharyngodon idella* kidney (CIK) cells were obtained from the China Center for Type Culture Collection (CCTCC) and were maintained at 28°C in 5% CO_2_ in medium 199 (M199) (Invitrogen) supplemented with 10% fetal bovine serum (FBS). The study utilized two subtypes of GCRV-I and GCRV-Ⅱ, GCRV-I (strain 873, group I), which was provided by Prof. Wu-han Xiao (Institute of Hydrobiology, Chinese Academy of Sciences), and GCRV-Ⅱ (strain HZ08, group Ⅱ), which was provided by Prof. Ya-Ping Wang (Institute of Hydrobiology, Chinese Academy of Sciences). Because GCRV-Ⅱ cannot cause a cytopathic effect (CPE) in CIK cells, but GCRV-I has the capacity, GCRV-I was used for virus infection in cells, whereas GCRV-Ⅱ was employed for viral challenge experiments in grass carp [45–47]. GCRV-I was propagated in CIK cells until a CPE was observed. Then, the harvested cell culture mixture containing GCRV-I was centrifuged at 4000 × *g* for 20 min to remove the cell debris, and the supernatant was stored at -80°C until use. The GCRV-Ⅱ used in the experiment was obtained as follows. Dead fish with typical symptoms of hemorrhagic diseases were collected and homogenized together with an equal volume of 0.75% saline. The mixture was subsequently centrifuged, and the supernatant was filtered through a 0.22 μm Millex filter (Millipore, SLGPR33RB).

### Plasmid construction and reagents

The sequence of *tollip* (GenBank accession number XM_051899887.1) was obtained from the National Center for Biotechnology Information (NCBI) website (http://www.ncbi.nlm.nih.gov/). Using the cDNA of the tissues from grass carp as a template, the open reading frame (ORF) of TOLLIP was amplified via polymerase chain reaction (PCR) and subsequently cloned and inserted into the pCMV-Myc, pCMV-HA, and pCMV-Tag2C vectors (Clontech). The ORFs of grass carp MAVS (KF366908.1), STING (JN786909.1), TBK1 (JN704345.1), ATG5 (MK635464.1), ATG14 (XM_051867309.1), Beclin1 (MG797682.1), p62 (MN311522.1), RAB5A (MF598473.1), and RAB7b (XM_051902268.1) were also subcloned and inserted into the pCMV-Myc, pCMV-HA, and pCMV-Tag2C vectors, respectively. For subcellular localization, the ORF of TOLLIP was inserted into the pEGFP-N3 (Clontech) and pCS2-mCherry vectors (Clontech), and the ORF of ATG5 was inserted into the pEGFP-N3. Truncated mutants of TOLLIP and microtubule-associated protein 1 light chain 3 (LC3, MG821471.1) were also subcloned and inserted into the pEGFP-N3 vector. The full-length and truncated mutants of STING were inserted into the pCS2-mCherry and pEGFP-N3 vectors. For promoter activity analysis, the IFN1pro-Luc construct was generated by inserting the corresponding 5′-flanking regulatory region of the IFN1 promoter (GU139255.1) into the pGL3-basic luciferase reporter vector (Promega). The IFN-stimulated response element (ISRE)-Luc plasmid in the pGL3-basic luciferase reporter vector was constructed as described previously [48]. The *Renilla* luciferase internal control vector (pRL-TK) was purchased from Promega. All the constructs were confirmed via DNA sequencing. Polyinosinic-polycytidylic acid (poly I:C) was purchased from Sigma-Aldrich and used at a final concentration of 1 µg/µl. MG132 (Cat. No. M7449), 3-methyladenine (3-MA) (Cat. No. M9281), and chloroquine (CQ) (Cat. No. C6628) were obtained from Sigma-Aldrich. Cycloheximide (CHX) (NSC-185) was obtained from Selleck.

### Transcriptomic analysis

Total RNA was extracted via TRIzol reagent according to the manufacturer’s protocol. RNA purity and quantification were evaluated via a NanoDrop 2000 spectrophotometer (Thermo Scientific). Transcriptome sequencing and analysis were conducted by OE Biotech Co., Ltd. (Shanghai). The raw sequencing data were submitted to the NCBI (GEO accession number: GSE296862).

### Luciferase activity assay

EPC cells were seeded in 24-well plates overnight and co-transfected with various constructs at a ratio of 5:5:5:1 (TOLLIP, STING, IFN1pro/ISRE-Luc, and pRL-TK expression vectors), and the empty vector was used to ensure equivalent amounts of total DNA in each well. Stimulation with poly I:C or GCRV-Ⅰ infection was performed at 24 h post-transfection. At 24 h post-stimulation, the cells were washed with phosphate-buffered saline (PBS) and lysed, and luciferase activity was measured using the Dual-Luciferase Reporter Assay System (Promega) according to the manufacturer’s instructions. Firefly luciferase activity was normalized to *Renilla* luciferase activity.

### Transient transfection and virus infection

Transient transfection of EPC cells, seeded in 6-well or 24-well plates, was performed using FishTrans (MeiSenTe Biotechnology, Guangdong, China) according to the manufacturer’s protocol. For the antiviral assay, EPC cells seeded in 24-well plates were transfected with 0.5 μg of TOLLIP-Flag or the empty vector. At 24 h post-transfection, the cells were infected with GCRV-Ⅰ [multiplicity of infection (MOI) = 0.001)]. After 48 h, the supernatant was transferred to CIK cells seeded in 24-well plates. Antiviral assays were performed as previously described [49]. For cell samples, 6-well plates were used, and EPC cells were transfected with 2 μg of TOLLIP-Flag or the empty vector. After 24 h, the cells were transfected with poly I:C or infected with GCRV-Ⅰ (MOI = 0.001) for 24 h, and total RNA was extracted to examine mRNA levels. For viral infection, the fish were anesthetized with methanesulfonate (MS-222) and intraperitoneally injected (i.p.) with 20 μL of M199 containing GCRV-II (2.97 × 10^3^ RNA copies/μL). For the control group, the fish were treated similarly and injected i.p. with 20 μL of PBS. The fish subsequently migrated into the aquarium, which contained new aquatic water.

### RNA extraction, reverse transcription, and quantitative real-time PCR (qPCR)

For fish tissue samples, at 48 h post-injection, all the fish were anesthetized with tricaine MS-222, dissected, and then the brain, heart, gill, head-kidney, kidney, spleen, liver, gut, gonad and skin were harvested, immediately frozen in liquid nitrogen and stored at -80°C for further qPCR assays. Total RNA was extracted with TRIzol reagent (Invitrogen). The genomic DNA was thoroughly digested, and first-strand cDNA was synthesized via a PrimeScript reverse transcription reagent kit (TaKaRa) according to the manufacturer’s instructions. qPCR was performed using the Fast SYBR Green PCR Master Mix (Yeasen) on a CFX96 real-time system (Bio-Rad). The PCR conditions were as follows: 95°C for 5 min, followed by 40 cycles of 95°C for 20 s, 60°C for 20 s, and 72°C for 20 s. All primers used for the qPCRs are shown in S1 Table, and the β*-*actin** gene was used as an internal control. The relative fold changes were calculated by comparing them to the corresponding controls using the 2^-ΔΔCt^ method. Three independent experiments were conducted for statistical analysis.

### RNA interference (RNAi)

The short hairpin RNAs (shRNAs) of *tollip* and *rab(5a/7b)* were designed with BLOCK-iT RNAi Designer and cloned and inserted into the pLKO.1-TRC cloning vector. For RNAi of *tollip*, EPC cells were seeded in 6-well plates overnight and transfected with 2 μg of *tollip shRNA* or the negative control (sh-NC). For RNAi of *rab5a/7b*, EPC cells were seeded in 6-well plates overnight and transfected with 1 μg shRNA of *rab5a/7b* or the negative control (sh-NC) and RAB5A/7b-Myc/HA for 24 h. After 24 h, the cells were harvested for the detection of RNAi effectiveness. All primers are shown in S1 Table.

### Co-immunoprecipitation (Co-IP) assay

For Co-IP experiments, EPC cells were seeded in 10 cm² dishes overnight and then transfected with 5 μg of each plasmid indicated in the figures. At 24 h post-transfection, the medium was carefully removed, and the cell monolayer was washed twice with 10 mL of ice-cold PBS. The cells were subsequently lysed in 1 mL of radioimmunoprecipitation (RIPA) lysis buffer [1% NP-40, 50 mmol/L Tris-HCl (pH 7.5), 150 mmol/L NaCl, 1 mmol/L EDTA, 1 mmol/L NaF, 1 mmol/L sodium orthovanadate (Na_3_VO_4_), 1 mmol/L phenyl-methylsulfonyl fluoride (PMSF), 0.25% sodium deoxycholate] containing a protease inhibitor cocktail (Yeasen) at 4°C for 1 h on a rocker platform. The cellular debris was removed by centrifugation at 12,000 × *g* for 15 min at 4°C. The supernatant was transferred to a fresh tube and incubated overnight at 4 °C with constant rotation in the presence of 20 µL of anti-Flag/HA/Myc affinity gel (Sigma-Aldrich). These samples were further analyzed by immunoblotting (IB). Immunoprecipitated proteins were collected by centrifugation at 5000 × *g* for 1 min at 4°C, washed three times with lysis buffer and resuspended in 100 μL of 1 × SDS sample buffer. The immunoprecipitates and whole-cell lysates (WCLs) were analyzed via IB with the indicated antibodies (Abs).

### Immunoblot analysis

The immunoprecipitates or WCLs were separated via 10% SDS-PAGE and transferred to polyvinylidene difluoride (PVDF) membranes (Millipore). The membranes were blocked for 1 h at room temperature in TBST buffer (25 mmol/L Tris-HCl, 150 mmol/L NaCl, 0.1% Tween 20, pH 7.5) containing 5% skim milk and then probed with the indicated primary Abs at an appropriate dilution overnight at 4°C. After being washed three times with TBST, the membranes were incubated with secondary Abs for 1 h at room temperature. After three additional washes with TBST, the membranes were stained with Immobilon Western chemiluminescent horseradish peroxidase (HRP) substrate (Millipore) and detected with a Touch Imager (e-Blot). Abs were diluted as follows: anti-β-actin (ABclonal, AC026) at a ratio of 1:10000, anti-Flag (Sigma-Aldrich, F1804) at a ratio of 1:3000, anti-HA (Covance, MMS-101R) at a ratio of 1:3000, anti-Myc (Santa Cruz Biotechnology, sc-40) at a ratio of 1:3000, and anti-LC3 (Abcam, ab48394) at a ratio of 1:1000. The indicated Abs of STING proteins at a ratio of 1:2000, TOLLIP proteins at a ratio of 1:100, and VP7 proteins at a ratio of 1:1000 were prepared by our laboratory.

### Immunofluorescence (IF)

EPC cells were plated onto glass coverslips in 6-well plates and infected with GCRV-Ⅰ (MOI = 0.001) for 24 h. Then, the cells were washed with PBS, fixed in 4% paraformaldehyde (PFA) at room temperature for 1 h and permeabilized with 0.2% Triton X-100 in ice-cold PBS for 15 min. The samples were blocked for 1 h at room temperature in PBS containing 2% bovine serum albumin (BSA; Sigma-Aldrich). After additional washes with PBS, the samples were incubated with anti-VP7 Ab in PBS containing 2% BSA for 2–4 h at 4°C. After three washes with PBS, the samples were incubated with a secondary Ab (goat anti-rabbit IgG (H + L) cross-conjugated, Alexa Fluor 488) (Thermo Scientific, A32731, 1:5000) in PBS containing 2% BSA for 1 h at room temperature. After additional washes with PBS, the cells were finally stained with 1 μg/ml 40,6-diamidino-2-phenylindole (DAPI; Beyotime Institute of Biotechnology) for 10 min in the dark at room temperature. Finally, the coverslips were washed and observed with a confocal microscope under a 20 × immersion objective (SP8, Leica).

### Staining of autophagic structures

DAPRed (autophagy detection kit, Dojindo, D677) was used following the supplier’s protocol outlined below. CIK cells were seeded overnight and then infected with GCRV-Ⅰ at an MOI of 0.001 for an additional 24 h. Subsequently, the cells were cultivated for 30 min in medium supplemented with 0.1 μM DAPRed. Canonical autophagy was examined with a 63 × immersion objective (SP8, Leica) after the staining medium was discarded.

### Transmission electron microscopy (TEM)

EPC and CIK cells were inoculated in 6-well plates and infected with GCRV-Ⅰ (MOI = 0.001) for 24 h. EPC cells were seeded in 6-well plates and transfected with the indicated plasmids for 24 h. For pretreatment, the cells were washed three times with PBS, trypsinized, and transferred to a 1.5 ml centrifuge tube. The cells were pelleted via centrifugation at 2000 × *g* for 5 min. The cell pellets were subsequently resuspended in 2.5% glutaraldehyde in 0.075 mol/L phosphate buffer (pH 7.4) for 4 h at 4°C for pre-fixation. Then, the cells were washed three times with a solution containing 0.075 mol/L phosphate and 0.19 mol/L sucrose for 15 min each, and post-fixed in 1% OsO_4_ in 0.24 mol/L phosphate buffer (pH 7.4) for 2 h. After being washed 3 times for 15 min each in 0.075 mol/L phosphate buffer and 0.19 mol/L sucrose buffer at 4°C, the cells were dehydrated using a graded series of ethanol and acetone, and then gradually infiltrated with epoxy resin. The samples were sequentially polymerized at 37°C overnight and then at 60°C for 48 h. Ultrathin sections (74 nm) were cut via a microtome (UC7, Leica) and mounted on copper slot grids. The sections were doubly stained with 3% uranyl acetate and lead citrate for 10 min and then observed under a transmission electron microscope (HT7700, Hitachi). Spleen tissues from control and virus-infected fish 2 days post-infection were dissected separately and resuspended in 2.5% glutaraldehyde in 0.075 mol/L phosphate buffer (pH 7.4) for 4 h at 4°C for pre-feeding, after which they were subjected to further treatments, such as those performed on cells.

### Fluorescence microscopy

EPC cells were plated onto coverslips in 6-well plates and transfected with the indicated plasmids for 24 h. Then, the cells were washed twice with PBS and fixed with 4% PFA for 1 h. After being washed three times with PBS, the cells were stained with DAPI for 15 min in the dark at room temperature. Finally, the coverslips were washed and observed with a confocal microscope under a 63 × oil immersion objective (SP8, Leica).

### LysoTracker staining

The transfected cells were incubated with 100 nM LysoTracker Red DND-99 (L-7528; Invitrogen) for 1 h at 28°C. The medium was aspirated, and the cells were washed twice quickly in PBS to remove unbound LysoTracker. The cells were then imaged under a confocal microscope using a 63 × oil immersion objective (SP8, Leica).

### Statistical analysis

For the dot plot, each dot represents one independent biological replicate. For the bar graph, one representative experiment of at least three independent experiments is shown, and each experiment was performed in triplicate. Unpaired Student’s *t*-test was used for statistical analysis. The data are expressed as the means ± standard errors of the means (SEMs). A *P*-value < 0.05 was considered statistically significant.

## Results

### 1. GCRV infection induces autophagic flux *in vivo* and in vitro

**Fig 1:**
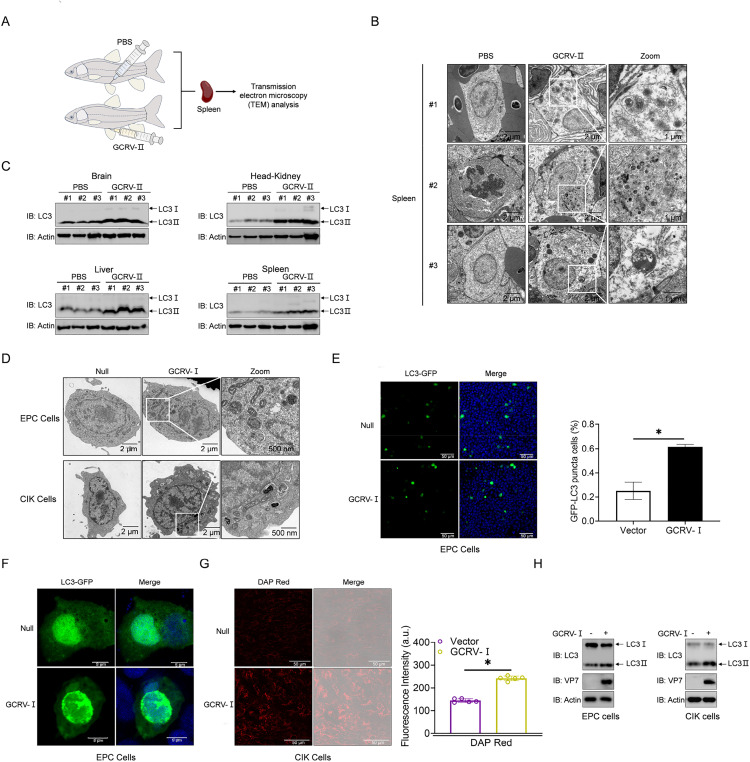
GCRV promotes autophagy *in vivo* and *in vitro.* (A) Schematic of grass carp tissue anatomy for TEM analysis. Grass carp were given i.p. PBS (20 μL/individual) or GCRV-Ⅱ (2.97 × 10^3^ RNA copies/μL, 20 μL/individual) for 48 h. Spleen tissues were isolated and subjected to TEM analysis. (B) TEM detection of autophagosome-like structures in the spleens of grass carp (n = 3 per group) given i.p. injections of PBS or GCRV-Ⅱ for 48 h. (C) IB analysis of LC3 in the brain, head-kidney, liver, and spleen of grass carp (n = 3 per group) given i.p. with PBS or GCRV-Ⅱ for 48 h. (D) TEM detection of autophagosome-like structures in untreated EPC and CIK cells or those infected with GCRV-Ⅰ (MOI = 0.001) for 24 h. (E and F) EPC cells were plated onto coverslips in 6-well plates, transfected with 2 μg of LC3-GFP, and infected with GCRV-Ⅰ (MOI = 0.001) 6 h later. Confocal microscopy detection of LC3-GFP for 24 h. (E) The cells were quantified under a 20 × immersion objective (SP8, Leica), and the data are expressed as the means ± SDs, n = 6. Statistical analysis was performed using Student’s *t*-test. Asterisks indicate significant differences from the control group (*P < 0.05). (F) Green signals represent overexpressed LC3 (original magnification 63 × ; oil immersion objective). (G) Fluorescence intensity detection of autophagosomes in CIK cells untreated or infected with GCRV-Ⅰ (MOI = 0.001) for 24 h, followed by treatment with DAPRed for 50 min. (H) IB analysis of LC3 in EPC and CIK cells, untreated or infected with GCRV-Ⅰ (MOI = 0.001) for 24 h. All experiments were repeated at least three times with similar results.

TEM analysis of spleen tissues from GCRV-Ⅱ-infected grass carp revealed prominent autophagic vesicles encapsulating cytoplasmic debris (Fig 1A and 1B). Concurrently, IB analysis demonstrated tissue-specific elevation of LC3-II conversion (a hallmark of autophagic flux) in brain, head-kidney, liver, and spleen tissues compared with that in uninfected controls (Fig 1C). Parallel *in vitro* TEM analysis of GCRV-Ⅰ-infected EPC and CIK cells confirmed the presence of double-membrane autophagosomes containing cytoplasmic material (Fig 1D). To further validate autophagosome formation, LC3-GFP-transfected EPC cells exhibited pronounced punctate fluorescence aggregation upon GCRV-Ⅰ infection (Fig 1E and 1F). Quantification of the fluorescence intensity of DAPRed, a specific autophagosome tracer, revealed a significant increase in GCRV-Ⅰ-infected CIK cells compared with controls (Fig 1G). IB analysis corroborated these findings, revealing significant LC3-Ⅱ upregulation in both EPC and CIK cells post-infection (Fig 1H). These multimodal observations collectively establish that GCRV infection triggers conserved autophagic activation at the organismal and cellular levels.

### 2. TOLLIP is upregulated in GCRV infection

**Fig 2:**
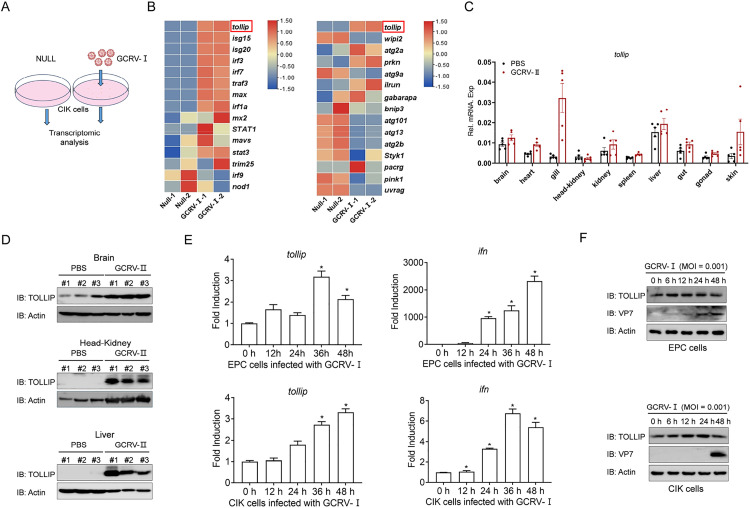
GCRV infection induces the upregulation of TOLLIP. (A) Schematic diagram of CIK cells transcriptome sequencing RNA extraction. Total RNA was extracted for transcriptome sequencing and analysis after CIK cells were left untreated or infected with GCRV-Ⅰ (MOI = 0.001) for 24 h. This illustration rendering portion of this work was supported by BioRender (https://www.biorender.com/). (B) Heatmap view of mRNA variations in CIK cells with or without GCRV-Ⅰ infection. (C) qPCR analysis of *tollip* mRNA in the brain, heart, gill, head-kidney, kidney, spleen, liver, gut, gonad, and skin of grass carp (n = 6 per group) given i.p. with PBS or GCRV-Ⅱ (2.97 × 10^3^ RNA copies/μL) for 48 h. (D) IB analysis of TOLLIP in the brain, liver and head kidney of grass carp (n = 3 per group) given i.p. with PBS or GCRV-Ⅱ for 48 h. (E) qPCR analysis of *ifn* and *tollip* mRNAs in EPC and CIK cells infected with GCRV-Ⅰ (MOI = 0.001) for the indicated times. (F) IB analysis of proteins in EPC and CIK cells infected with GCRV-Ⅰ (MOI = 0.001) for the indicated times. All experiments were repeated at least three times with similar results.

To identify potential molecules associated with GCRV-induced autophagy, integrated transcriptomic profiling of GCRV-Ⅰ-infected CIK cells revealed significant changes in immune response genes and autophagy-related genes, with *tollip* emerging as a prominently induced candidate among immune- and autophagy-related genes, suggesting its potential involvement in GCRV-Ⅰ-induced autophagy (Fig 2A and 2B). Subsequent spatiotemporal expression analyses revealed coordinated regulation between antiviral responses and TOLLIP expression. Compared with those of the uninfected controls, the *in vivo* results revealed the upregulation of progressive *tollip* transcripts in multiple tissues, particularly the gill, kidney, and liver, of GCRV-Ⅱ-infected grass carp. Moreover, IB analysis confirmed the corresponding elevation of the TOLLIP protein in infected tissues (brain, head kidney, and liver) (Fig 2C and 2D). *In vitro* infection models corroborated these findings; the mRNAs of *tollip* were upregulated over time in both CIK cells and EPC cells, whereas concurrent induction of *ifn* transcripts confirmed active IFN signalling (Fig 2E). Furthermore, an accumulation of TOLLIP protein was observed, reaching its maximum at 24 h and subsequently decreasing through 48 h in both CIK and EPC cells (Fig 2F). This conserved upregulation pattern across the organismal and cellular levels, spanning transcriptional and translational regulation, establishes TOLLIP as a responsive element during GCRV infection.

### 3. TOLLIP inhibits GCRV/poly I:C-induced IFN expression

**Fig 3:**
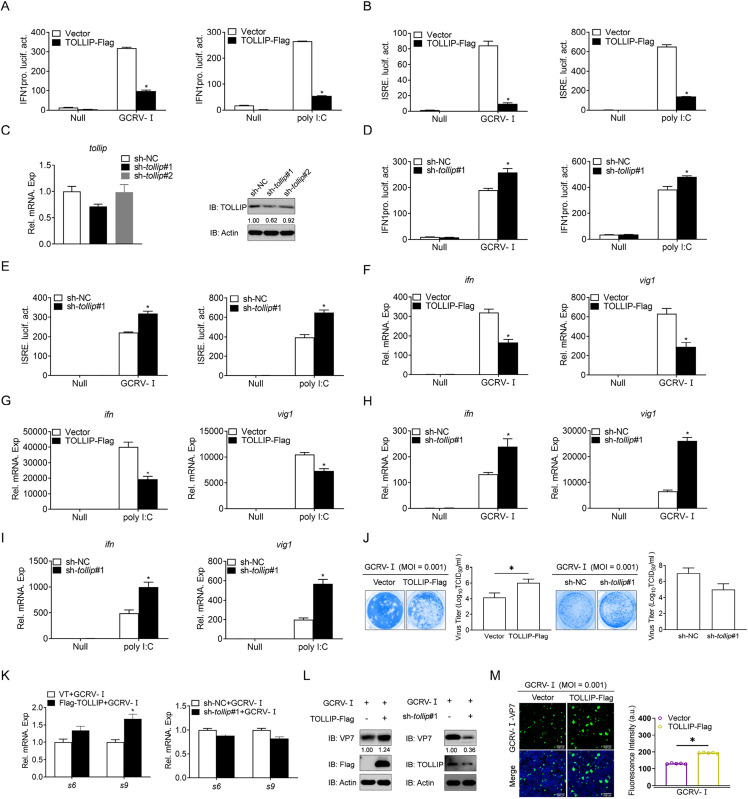
TOLLIP negatively regulates IFN expression and promotes GCRV replication. (A and B, D and E) Luciferase activity of IFN1pro and ISRE in EPC cells transfected with the indicated plasmids for 24 h and then untreated or infected with GCRV-Ⅰ (MOI = 0.001) or transfected with poly I:C (0.5 μg) for 24 h before luciferase assays. (C) qPCR and IB analysis of TOLLIP in EPC cells transfected with the indicated plasmids for 24 h. (F-I) qPCR analysis of *ifn* and *vig1* in EPC cells transfected with the indicated plasmids for 24 h and then untreated or infected with GCRV-Ⅰ (MOI = 0.001) or transfected with poly I:C (2 μg) for 24 h. (J) EPC cells were transfected with the indicated plasmids for 24 h, infected with GCRV-Ⅰ (MOI = 0.001) for 48 h, and then the supernatant was transferred to CIK cells. Plaque assays for viral titers were performed 24-48 h later. (K and L) qPCR and IB analysis of GCRV-Ⅰ genes in EPC cells transfected with the indicated plasmids for 24 h, followed by GCRV-Ⅰ (MOI = 0.001) challenge for 24 h. IB was subjected to grayscale analysis, with grayscales calculated relative to actin levels and normalized to the protein level of control cells, which was set to 1. (M) IF analysis of GCRV-Ⅰ-VP7 in EPC cells transfected with the indicated plasmids for 24 h, followed by challenge with GCRV-Ⅰ (MOI = 0.001) for 24 h. The fluorescence intensity (arbitrary unit, a.u.) was recorded with LAS X software, and the data are expressed as the means ± SDs, n = 5. All experiments were repeated at least three times with similar results.

To clarify the regulatory role of TOLLIP in antiviral immunity, we first assessed its impact on IFN activation in EPC cells. Given that poly I:C is a viral RNA mimic and that grass carp type I IFNs include four members (IFN1-IFN4), only IFN1 can be activated by poly I:C [50]. Consequently, the promoter of IFN1 (IFN1pro) was used in subsequent assays in EPC cells. As shown in Fig 3A, both GCRV-Ⅰ infection and poly I:C stimulation robustly activated IFN1pro, but this activation was significantly attenuated by TOLLIP overexpression. The ISRE motif is considered the binding site for ISGs in response to transcription factors [51]. The activation of ISRE induced by GCRV-Ⅰ or poly I:C was also suppressed by TOLLIP (Fig 3B). An effective sh-*tollip* was produced and identified at the transcriptional and protein levels, and the knockdown of *tollip* facilitated both the IFN promoter and ISRE activity (Fig 3C-3E). In addition, overexpression of TOLLIP inhibited GCRV-Ⅰ/poly I:C-stimulated transcription of *ifn* and *vig1* at the mRNA level, whereas knockdown of *tollip* had the opposite effect (Fig 3F-3I). In the antiviral capacity assays, TOLLIP overexpression exacerbated GCRV-Ⅰ-induced CPE in CIK cells and increased the viral load, whereas *tollip* knockdown resulted in a decrease in the viral load (Fig 3J). Moreover, TOLLIP promoted the upregulation of GCRV-Ⅰ-related genes, and *tollip* knockdown had the opposite effect (Fig 3K). IB analysis of the GCRV-Ⅰ structural protein VP7 confirmed these findings, revealing elevated VP7 levels in TOLLIP-overexpressing cells and reduced accumulation in *tollip* knockdown cells (Fig 3L). IF analysis further corroborated these results, with TOLLIP-overexpressing cells exhibiting stronger VP7 signals than control cells (Fig 3M). Collectively, these data establish TOLLIP as a negative regulator of antiviral responses, operating through the suppression of IFN expression to facilitate GCRV replication.

### 4. TOLLIP interacts with STING

**Fig 4:**
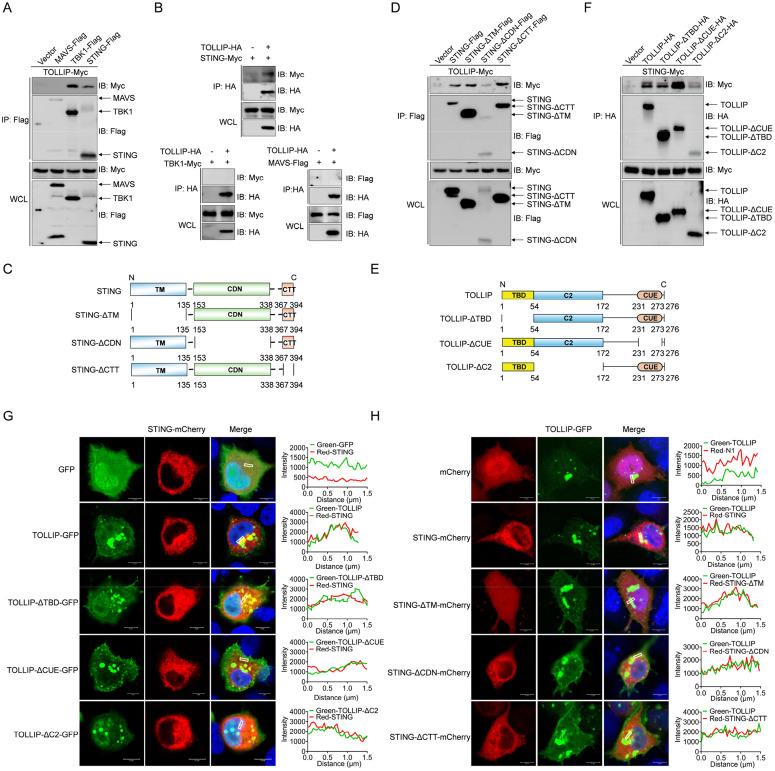
TOLLIP interacts with STING. (A and B) IB analysis of WCLs and proteins immunoprecipitated with anti-Flag or anti-HA Ab-conjugated agarose beads from EPC cells transfected with the indicated plasmids for 24 h. (C) Schematic representation of full-length STING and its deletion mutants. (D and F) IB analysis of WCLs and proteins immunoprecipitated with anti-Flag or anti-HA Ab-conjugated agarose beads from EPC cells transfected with the indicated plasmids for 24 h. (E) Schematic representation of full-length TOLLIP and its deletion mutants. (G and H) Confocal microscopy of TOLLIP and STING or its deletion mutants in EPC cells transfected with the indicated plasmids for 24 h. The coefficient of colocalization was determined through qualitative analysis of the fluorescence intensity in the selected area of Merge. All experiments were repeated at least three times with similar results.

To elucidate the molecular mechanism underlying TOLLIP-mediated suppression of IFN responses, we investigated the interaction of TOLLIP with key components of RLR signalling cascades. Co-IP assays in EPC cells transfected with Myc-tagged TOLLIP and FLAG-tagged RLR pathway proteins (MAVS, TBK1, and STING) revealed the selective binding of TOLLIP to TBK1 and STING but not to MAVS (Fig 4A). Reciprocal Co-IP confirmed the bidirectional interaction between TOLLIP and STING, whereas TOLLIP-TBK1 and TOLLIP-MAVS binding were unidirectional (Fig 4B). To map the STING interaction domain, we generated three truncated deletion mutants: STING-ΔTM (lacking the N-terminal TM region), STING-ΔCDN (lacking the CDN region), and STING-ΔCTT (lacking the CTT region) (Fig 4C). Co-IP assays demonstrated that all the mutants retained their TOLLIP-binding capacity, indicating that distributed interactions may interact across the structural domains of STING (Fig 4D). We subsequently investigated which region of TOLLIP is essential for its interaction with STING. To address this issue, three TOLLIP truncated deletion mutants were generated: TOLLIP-ΔTBD (lacking the TBD domain), TOLLIP-ΔCUE (lacking the CUE domain), and TOLLIP-ΔC2 (lacking the C2 domain) (Fig 4E). As shown in Fig 4F, STING interacted with the full-length TOLLIP as well as truncations of the TOLLIP domain. Next, the subcellular localization of TOLLIP and STING was monitored in EPC cells. Confocal microscopy analysis revealed that the TOLLIP and its deletion-enhanced GFP signals were distributed mainly in the cytoplasm in a plaque-like manner and partially colocalized with the red signals from STING, whereas the signals of STING and its mutants also partially overlapped with those of TOLLIP in the cytoplasm (Fig 4G and 4H). These results demonstrate that TOLLIP specifically binds to STING, a key molecule of the RLR pathway.

### 5. TOLLIP degrades STING in a dose-dependent manner

**Fig 5:**
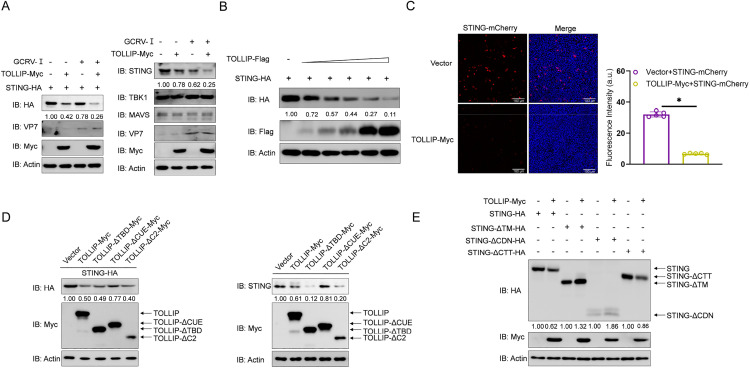
TOLLIP degrades STING. (A and B) IB analysis of proteins in EPC cells transfected with the indicated plasmids for 24 h, followed by treatment with or without GCRV-Ⅰ (MOI = 0.001) for 24 h. (C) Fluorescence intensity detection of STING-mCherry in EPC cells transfected with the indicated plasmids for 24 h. (D-E) IB analysis of proteins in EPC cells transfected with the indicated plasmids for 24 h. All experiments were repeated at least three times with similar results.

The above results revealed that TOLLIP is associated with STING, so it is necessary to study the relationship between TOLLIP and STING. At the protein level, TOLLIP expression effectively promoted endogenous and exogenous STING degradation in both normal and GCRV-Ⅰ-stimulated states, while has no effect on TBK1 and MAVS in either case (Fig 5A). Furthermore, this degradation effect exhibited a clear dose-response relationship, with a progressive increase in STING reduction observed with increasing TOLLIP expression levels (Fig 5B). Moreover, overexpression of TOLLIP substantially diminished the fluorescence intensity of STING-mCherry (Fig 5C). We further explored the degradation of STING by different structural domains of TOLLIP. While both the ΔC2 and ΔTBD mutants of TOLLIP maintained full degradation activity, the ΔCUE mutant completely lost a great part of its capacity to reduce STING levels, indicating essential roles for the CUE domain in mediating STING degradation (Fig 5D). Reciprocal studies of the structural determinants of STING have shown that CTT domain deletion mutants can still be degraded by TOLLIP. However, deletion of either the TM or CDN domains was largely unaffected by TOLLIP (Fig 5E). These results suggest that TOLLIP degrades the TM and CDN regions of STING primarily through the structural domain of the CUE.

### 6. TOLLIP mediates the autophagy-lysosome-dependent degradation of STING

**Fig 6:**
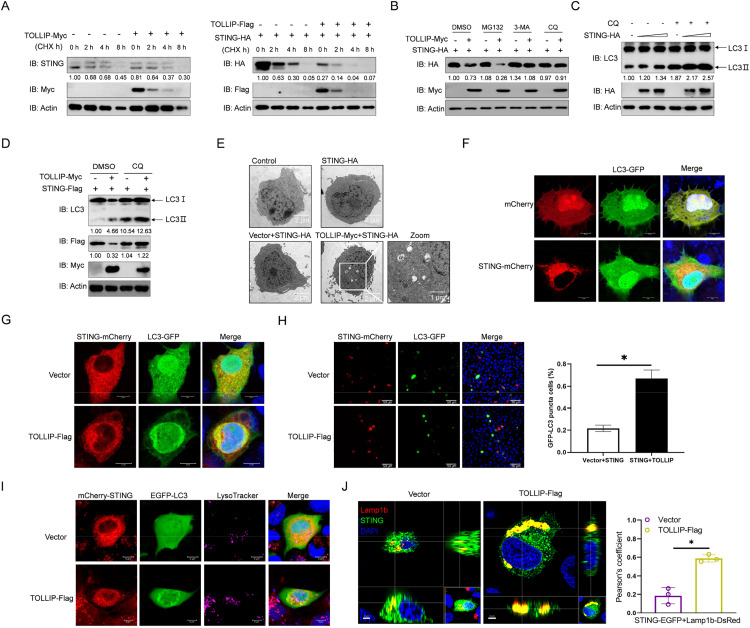
TOLLIP degrades STING via the autophagy-lysosome-dependent pathway. (A) IB analysis of proteins in EPC cells transfected with the indicated plasmids for 18 h, followed by treatment with CHX (50 mM) for the indicated times. (B) IB analysis of proteins in EPC cells transfected with the indicated plasmids for 18 h, followed by treatment with MG132 (10 μM), 3-MA (2 mM) or CQ (50 μM) for 6 h. (C and D) IB analysis of proteins in EPC cells transfected with the indicated plasmids for 18 h, followed by treatment with CQ (50 μM) for 6 h. (E) TEM detection of autophagosome-like structures in EPC cells transfected with the indicated plasmids for 18 h, followed by treatment with CQ (50 μM) for 6 h. (F-H) EPC cells were plated onto coverslips in 6-well plates and cotransfected with the indicated plasmids for 18 h, followed by treatment with CQ (50 μM) for 6 h. The cells were fixed and observed via confocal microscopy. Red signals represent overexpressed STING protein, and green signals represent overexpressed LC3 (original magnification 63 × , oil immersion objective). (H) The cells were quantified under a 20 × immersion objective (SP8, Leica), and the data are expressed as the means ± SDs, n = 5. Statistical analysis was performed using Student’s *t*-test. Asterisks indicate significant differences from the control group (*P < 0.05). (I) EPC cells were plated onto coverslips in 6-well plates and cotransfected with the indicated plasmids for 18 h, followed by treatment with CQ (50 μM) for 6 h. The cells were then stained with LysoTracker (100 nM) for 1 h and subjected to confocal microscopy analysis. The purple signals represent LysoTracker, the red signals represent overexpressed STING protein, and the green signals represent overexpressed LC3 (original magnification 63 × ; oil immersion objective). (J) EPC cells were plated onto coverslips in 6-well plates and cotransfected with the indicated plasmids for 18 h, followed by treatment with CQ (50 μM) for 6 h. The cells were fixed and observed via confocal microscopy. Red signals represent overexpressed Lamp1b, and green signals represent overexpressed STING (original magnification 100 × ; oil immersion objective). The panel displays a three-dimensional rendering of a representative field generated using Imaris software, and the Pearson correlation coefficient from three independent replicate experiments was calculated for statistical analysis. All experiments were repeated at least three times with similar results.

To further investigate the stability of the STING protein in the presence of TOLLIP, we performed CHX chase experiments and found that overexpression of TOLLIP led to accelerated degradation of both endogenous and exogenous STING (Fig 6A). To identify the degradation pathway involved, pharmacological inhibition experiments were conducted in EPC cells co-expressing STING and TOLLIP. As shown in Fig 6B, the proteasome inhibitor MG132 failed to rescue STING degradation, but the autophagy pathway inhibitors 3-MA and CQ restored STING protein levels, suggesting that TOLLIP degrades STING through an autophagy-lysosome-dependent pathway. To further demonstrate that TOLLIP degrades STING through the autophagy-lysosome-dependent pathway, we examined the changes in LC3-Ⅱ in the context of overexpressing STING alone and co-transfecting TOLLIP and STING. The results revealed that there was no LC3-Ⅱ accumulation in STING-only overexpressing cells, but the expression of LC3-Ⅱ gradually increased after TOLLIP and STING co-transfection (Fig 6C and 6D). TEM analysis revealed more autophagosome-like vesicles containing cytoplasmic contents in cells co-overexpressing TOLLIP and STING than in those overexpressing STING alone (Fig 6E). Confocal microscopy revealed that the aggregation of LC3-GFP was relatively low when STING was overexpressed alone, whereas co-expressing TOLLIP with STING resulted in increased patchy aggregation of LC3-GFP (Fig 6F-6H). To assess lysosomal dynamics during TOLLIP-mediated STING degradation, we monitored lysosomal localization. Upon TOLLIP overexpression, STING and lysosomes co-aggregated, with lysosomes predominantly accumulating within LC3-GFP-labeled autophagic structures (Fig 6I). Further analysis using Lamp1b-DsRed (lysosomal marker) and STING-GFP revealed significantly enhanced colocalization under TOLLIP overexpression via confocal microscopy, indicating TOLLIP promotes STING trafficking to lysosomes (Fig 6J). Collectively, these data demonstrate that TOLLIP degrades STING through the autophagy-lysosome pathway.

### 7. ATG5 is essential for TOLLIP-mediated autophagic degradation of STING

**Fig 7:**
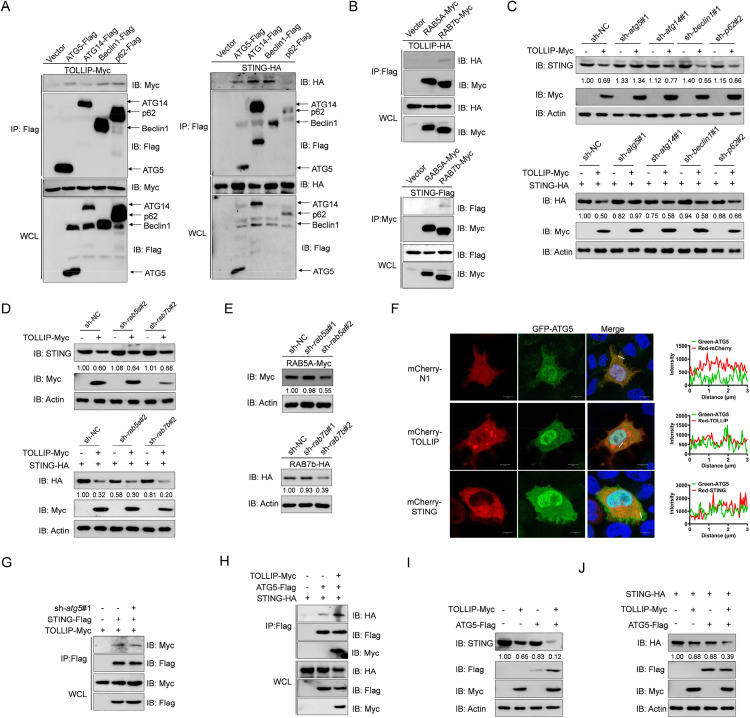
ATG5 is essential for TOLLIP-induced degradation of STING. (A and B) IB analysis of WCLs and proteins immunoprecipitated with anti-Flag or anti-HA Ab-conjugated agarose beads from EPC cells transfected with the indicated plasmids for 24 h. (C-E) IB analysis of proteins in EPC cells transfected with the indicated plasmids for 24 h. (F) Confocal microscopy of TOLLIP and STING with ATG5 in EPC cells transfected with the indicated plasmids for 24 h. The coefficient of colocalization was determined through qualitative analysis of the fluorescence intensity in the selected area of Merge. (G and H) IB analysis of WCLs and proteins immunoprecipitated with anti-Flag Ab-conjugated agarose beads from EPC cells transfected with the indicated plasmids for 24 h. (I and J) IB analysis of proteins in EPC cells transfected with the indicated plasmids for 24 h. All experiments were repeated at least three times with similar results.

To delineate the mechanism of TOLLIP-mediated autophagic degradation of STING, we cloned and screened key autophagy regulators. Co-IP assays demonstrated that TOLLIP interacts with ATG5, ATG14, Beclin1, p62, and RAB7b, while STING binds ATG5, ATG14, Beclin1, and RAB7b (Fig 7A and 7B). Critically, IB analysis showed that only *atg5* knockdown attenuated TOLLIP-mediated degradation of both endogenous and exogenous STING, depletion of other autophagy-related proteins basically had no effect (Fig 7C and 7D). Knockdown efficiencies for *atg5*, *atg14*, *beclin1*, and *p62* were validated in prior studies, with *rab5a* and *rab7b* efficiencies confirmed in Fig 7E [34,52–54]. These findings implicated ATG5 in TOLLIP-driven STING degradation. Confocal microscopy analysis revealed that the green signal of ATG5 was distributed throughout the cell and partially overlapped with the red signals of TOLLIP or STING (Fig 7F). We therefore examined the role of ATG5 in the TOLLIP-STING interaction. As predicted, knockdown *atg5* weakened TOLLIP-STING binding, meanwhile, TOLLIP overexpression augmented ATG5-STING interaction (Fig 7G and 7H). Furthermore, ATG5 overexpression substantially potentiated TOLLIP-mediated degradation of endogenous and exogenous STING (Fig 7I and 7J). Taken together, these results indicate that ATG5 is indispensable for TOLLIP-directed autophagic degradation of STING.

### 8. TOLLIP antagonizes STING-dependent antiviral signalling

**Fig 8:**
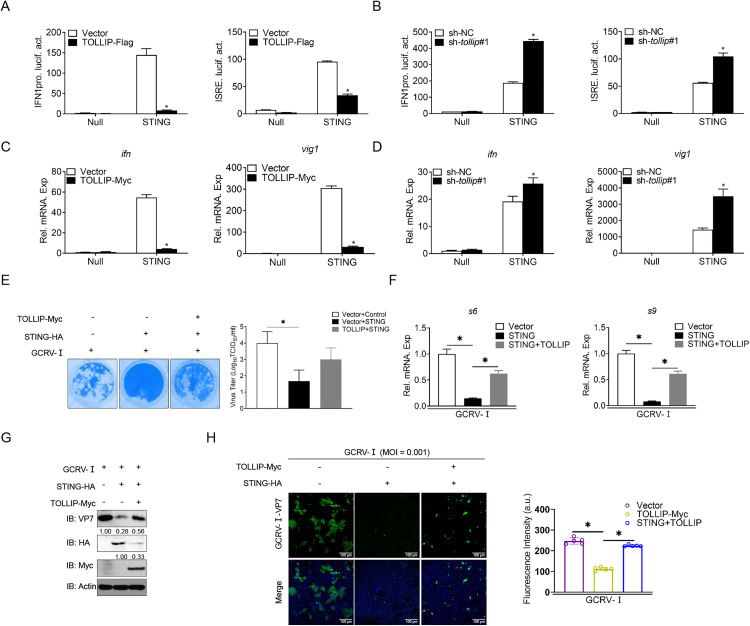
TOLLIP inhibits the antiviral function of STING. (A and B) Luciferase activity of IFN1pro and ISRE in EPC cells transfected with the indicated plasmids for 24 h. (C and D) qPCR analysis of *ifn* and *vig1* in EPC cells transfected with the indicated plasmids for 24 h. (E) EPC cells were transfected with the indicated plasmids for 24 h, followed by a 48 h challenge with GCRV-Ⅰ (MOI = 0.001). The supernatant was then transferred to CIK cells, and plaque assays were performed to determine viral titers 24 to 48 h later. (F) qPCR analysis of *s6* and *s9* in EPC cells transfected with the indicated plasmids for 24 h and then infected with GCRV-Ⅰ (MOI = 0.001) for 24 h. (G) IB analysis of proteins in EPC cells transfected with the indicated plasmids for 24 h and then infected with GCRV-Ⅰ (MOI = 0.001) for 24 h. (H) IF analysis of VP7 in EPC cells transfected with the indicated plasmids for 24 h, followed by GCRV-Ⅰ (MOI = 0.001) challenge for 24 h. All experiments were repeated at least three times with similar results.

To determine whether TOLLIP functionally impairs STING-mediated immunity, we first assessed its impact on STING-driven IFN responses. Luciferase assays demonstrated that TOLLIP overexpression significantly inhibited STING-mediated IFN activation, whereas *tollip* knockdown significantly enhanced STING-induced IFN expression (Fig 8A and 8B). Consistent results were obtained at the mRNA level (Fig 8C and 8D). CPE quantification revealed that STING overexpression reduced GCRV-Ⅰ-induced cell damage. In contrast, TOLLIP co-expression abolished this protective effect (Fig 8E). Functional validation in GCRV-Ⅰ-infected cells revealed that STING overexpression reduced viral *s6* and *s9* transcript levels. This effect was significantly reversed by TOLLIP co-expression (Fig 8F). At the protein level, TOLLIP reversed the STING-mediated decrease in VP7 expression (Fig 8G). IF analysis further confirmed these results, with TOLLIP counteracting the STING-driven suppression of VP7 protein accumulation (Fig 8H). Collectively, these data establish TOLLIP as a potent suppressor of STING-dependent antiviral immunity.

## Discussion

**Fig 9:**
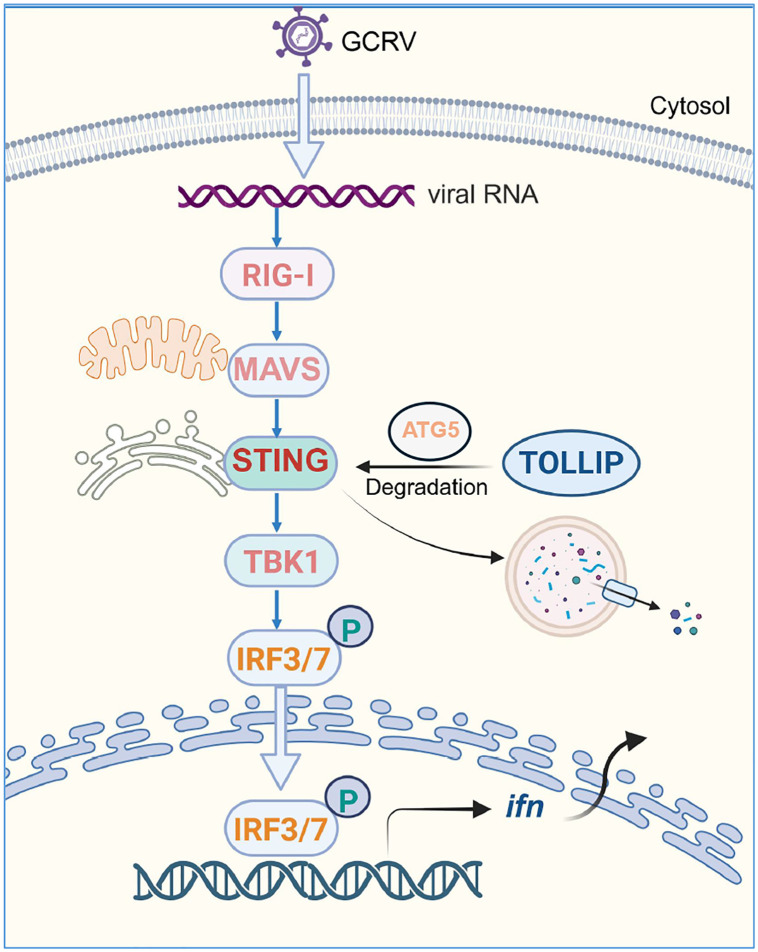
Schematic representation illustrating that fish TOLLIP negatively regulates STING-induced IFN expression. Upon GCRV infection, fish RIG-I senses viral RNA and interacts with MAVS, leading to the recruitment of TBK1, which phosphorylates STING and IRF3/7 and then induces IFN production. TOLLIP binds ATG5 to degrade STING via an autophagy-lysosome-dependent pathway, thereby inhibiting IFN expression. This illustration rendering portion of this work was supported by BioRender (https://www.biorender.com/).

Although the IFN system serves as a robust antiviral defence mechanism in fish, its dysregulation can lead to detrimental consequences, including severe autoimmune disorders and chronic inflammatory responses. Consequently, precise regulation of IFN homeostasis through balanced immune modulation is crucial for maintaining host fitness [55,56]. In this study, we elucidated the autophagy-related protein TOLLIP as a critical negative regulator of IFN homeostasis. Mechanistically, TOLLIP interacts with STING, a central adaptor of the RLR signalling pathway, and targets it for autophagic degradation. Moreover, ATG5 binds to both STING and TOLLIP, playing a crucial role in TOLLIP-mediated degradation of STING, eventually suppressing IFN production (Fig 9). Our findings delineate a previously unrecognized immunoregulatory axis in fish while expanding the functional repertoire of TOLLIP beyond its role as a canonical autophagy receptor to include IFN signalling suppression through targeted STING degradation.

While autophagy represents an evolutionarily conserved antiviral mechanism through elimination of intracellular pathogens, increasing evidence reveals its paradoxical exploitation by viruses as a replication-promoting strategy. This evolutionary arms race has led to the development of sophisticated viral countermeasures that repurpose the autophagic machinery for immune evasion and enhancement of pathogenesis. Substantial evidence has demonstrated that certain viruses actively induce autophagic flux to establish proviral microenvironments. For example, swine acute diarrhea syndrome coronavirus (SADS-CoV) envelope protein E induces signal transduction and activator of transcription 2 (STAT2) degradation via the autophagic-lysosomal pathway, effectively suppressing IFN-I signalling [57]. Through such molecular piracy, viruses manipulate the host autophagic machinery to counteract finely tuned immune defences, achieving optimized immune escape. In fish, herpesvirus KLP utilizes Beclin1 to selectively degrade STING via autophagic pathway [58]. And our previous study revealed that GCRV VP4 recruits TOLLIP to achieve immune escape [59]. In this study, we found that TOLLIP orchestrates the autophagic degradation of STING through a direct physical interaction, thereby suppressing IFN-mediated antiviral responses and maintaining immune equilibrium. Collectively, these data establish TOLLIP as a dual-function modulator of fish antiviral immunity, balancing STING-dependent immune homeostasis against inadvertent proviral consequences during GCRV challenge.

Extensive studies reveal conserved TOLLIP-STING interactions. Human TOLLIP stabilizes resting-state STING while acting as an autophagy receptor for viral proteins, facilitating infection-induced STING degradation [40]. For example, pseudorabies virus (PRV) UL38 recruits TOLLIP to degrade STING via autophagy, suppressing cGAS-STING signaling [60]. Murine TOLLIP deficiency enhances mitophagy and reduces STING activation during influenza A virus infection [61]. While TOLLIP universally participates in autophagy coordination, its specific roles exhibit species-specific adaptation. In mice, TOLLIP acts as a key coordinator for Parkin-dependent lysosomal sorting of damaged mitochondria [62]. In Avian, it serves as an autophagy receptor. For example, MARCH6 recruits TOLLIP to degrade Tembusu virus (TMUV) NS5 [63]. In fish, TOLLIP promotes STING destabilization through autophagy-lysosome-dependent pathway. Collectively, these findings suggest that while TOLLIP participates universally in selective autophagy pathways, its precise mechanistic roles and regulatory contexts vary across species.

Mammalian TOLLIP stabilizes STING to maintain tissue immune homeostasis, whereas fish TOLLIP promotes STING autophagic degradation to prevent IFN overproduction. This divergence is probably indicative of lineage-specific adaptations that have been shaped by distinct environmental and physiological pressures. First, it is important to consider that aquatic ecosystems expose fish to elevated pathogen densities and frequent viral challenges. The rapid termination of STING signalling via TOLLIP-mediated degradation may be a conserved energy-conserving mechanism in poikilothermic organisms with limited metabolic scope. In contrast, terrestrial mammals prioritize immune surveillance in response to sporadic pathogen encounters and the avoidance of autoimmunity by stabilizing the STING pathway [35]. Second, fish may employ unidentified STING stabilizers to ensure basal immune readiness, thereby offsetting constitutive degradation. In contrast, mammals employ parallel degradative pathways to reduce reliance on TOLLIP for STING turnover. This evolutionary plasticity underscores TOLLIP’s role as a lineage-specific “toggle” that fine-tunes antiviral responses across vertebrates. Subsequent studies should be directed toward investigating whether this divergence extends to other ectothermic vertebrates (e.g., amphibians and reptiles).

Our results establish the CUE domain as essential for TOLLIP-mediated STING degradation. As established in prior studies, the CUE domain of TOLLIP is indispensable for its function in selective autophagy. In humans, this domain recruits ubiquitinated substrates to LC3-decorated autophagosomes via LIR motifs [64]. Furthermore, it has been demonstrated that this process enhances the affinity for mono- and polyubiquitin chains [36]. Evolutionary conservation is evidenced by yeast Cue5, a ubiquitin-Atg8 adaptor mitigating aggregation-prone protein cytotoxicity [36]*.* Critically, our data revealed that the CUE domain is essential for STING degradation despite being dispensable for STING binding (Figs 4F and 5D). This finding is consistent with the established canonical ubiquitin-recognition function of the CUE domain, suggesting that it facilitates the ubiquitin-dependent recruitment of STING to the autophagy machinery. Furthermore, an amino acid sequence comparison confirmed that grass carp TOLLIP-CUE shares 58.64% similarity with human and yeast orthologs. Consequently, we hypothesize that grass carp TOLLIP functions as an adaptor that couples ubiquitinated STING to LC3 via its LIR motif. Future work will define TOLLIP-recognized STING ubiquitination signatures and validate this adaptor model.

In conclusion, this study establishes fish TOLLIP as a pleiotropic regulator that coordinates autophagic processes and antiviral immunity. During GCRV infection, TOLLIP undergoes significant transcriptional and translational upregulation to suppress host defence, direct inhibition of virus-induced IFN production and autophagy-lysosome-dependent degradation of STING through the manipulation of ATG5, ultimately facilitating viral replication. This study provides the first evidence that fish TOLLIP immunomodulates the host by targeting STING. Future investigations should address the structural determinants underlying the species-specific functional switching of TOLLIP and evaluate its potential as a therapeutic target for modulating antiviral immunity in aquaculture species.

## Supporting information

S1 Table Primers used in this study. (XLSX)
